# The LET Procedure for Prosthetic Myocontrol: Towards Multi-DOF Control Using Single-DOF Activations

**DOI:** 10.1371/journal.pone.0161678

**Published:** 2016-09-08

**Authors:** Markus Nowak, Claudio Castellini

**Affiliations:** Robotics and Mechatronics Center, DLR — German Aerospace Center, Wessling, Germany; Duke University, UNITED STATES

## Abstract

Simultaneous and proportional myocontrol of dexterous hand prostheses is to a large extent still an open problem. With the advent of commercially and clinically available multi-fingered hand prostheses there are now more independent degrees of freedom (DOFs) in prostheses than can be effectively controlled using surface electromyography (sEMG), the current standard human-machine interface for hand amputees. In particular, it is uncertain, whether several DOFs can be controlled simultaneously and proportionally by exclusively calibrating the intended activation of single DOFs. The problem is currently solved by training on all required combinations. However, as the number of available DOFs grows, this approach becomes overly long and poses a high cognitive burden on the subject. In this paper we present a novel approach to overcome this problem. Multi-DOF activations are artificially modelled from single-DOF ones using a simple linear combination of sEMG signals, which are then added to the training set. This procedure, which we named LET (Linearly Enhanced Training), provides an augmented data set to any machine-learning-based intent detection system. In two experiments involving intact subjects, one offline and one online, we trained a standard machine learning approach using the full data set containing single- and multi-DOF activations as well as using the LET-augmented data set in order to evaluate the performance of the LET procedure. The results indicate that the machine trained on the latter data set obtains worse results in the offline experiment compared to the full data set. However, the online implementation enables the user to perform multi-DOF tasks with almost the same precision as single-DOF tasks without the need of explicitly training multi-DOF activations. Moreover, the parameters involved in the system are statistically uniform across subjects.

## Introduction

From a mechatronic point of view, commercially available hand prostheses have made great advances in the past decade. In particular, Otto Bock’s *Michelangelo*, Touch Bionics’ *i-LIMB Ultra Revolution*, Vincent Systems’ *Vincent Hand Evolution2* and RSL Steeper’s *BeBionic* all allow individual finger motions of various degrees of motion and dexterity. This is due to the presence of several independent motors, potentially allowing for complex grip patterns, proportional force control and independent control of individual fingers. These devices are not yet as advanced as research prototypes of mechatronic hand, as they must comply with a number of constraints (weight, cost, robustness and power consumption). For example, these prostheses must be worn for up to 12 hours a day.

One of the main challenges of rehabilitation robotics is to enable the user with proper control of the hand prosthesis. Recent studies [1–3] indicate that the lack of proper control is one of the main factors of rejection.

Therefore, new ways of gathering and processing bodily signals, in the form of novel Human-Machine Interfaces (HMIs) [4–7], are being actively sought for, since the existing approach, in which two surface electromyography (sEMG) electrodes are used to control a 1-degree of freedom (DOF) prosthetic gripper, no longer suffices. To extend the control solution to prostheses with a higher number of DOFs researchers have focused on higher numbers of sEMG electrodes and different types of signals [4, 5]. At the same time, machine-learning (ML) approaches are being employed to interpret the signals, to enforce natural detection of the *intent* of the user.

However, no such solution is yet used in clinical applications or in daily life. The main problem remains one of *reliability*; that is avoiding false positives and negatives detected by the ML system, which could have catastrophic consequences. From the patient’s point of view [2] it is still preferable to have a simple and reliable gripper, rather than a multi-DOF mechanism, which will occasionally drop a shopping bag or worse. Nowadays, there are more DOFs than the user can reliably control. *The control system has become the bottleneck* to high-DOF prosthesis performance.

Along this line of investigation, in this paper we propose an enhancement to ML-based HMIs for hand prosthetic control, leading to a potentially *finer* and *simpler* control of many DOFs simultaneously and proportionally (s/p). As the number of DOFs of a prosthesis grows, it becomes increasingly important that the calibration procedure stays manageable for the user, while ensuring that each DOF can be properly controlled. Conventionally, to enforce a set of grasping patterns (e.g., power grasp, pinch grip, lateral grip, etc.), the user must produce appropriate signal patterns for each grasp. These signal patterns represent coordinated activations of many DOFs at the same time. This results in control limitations, as it ignores the potential to independently control each motor of the prosthesis. At the same time, it is not feasible to have the user produce reliable patterns for the activation of each DOF individually, as well as for each combination of DOFs. In this case the number of patterns grows exponentially with the number of DOFs, as does the required training time and effort.

Our main goal is to provide s/p control over both single- and multi-DOF activations, possibly also involving the DOFs of a prosthetic wrist (pronation, supination, radial abduction). This poses a great challenge: a system composed of a multi-finger hand prosthesis and a 2-DOF wrist can already have up to 8 DOFs. Some way of automatically combining single-DOF activations into multi-DOF ones is therefore desirable. This paper shall introduce and discuss, along with experimental findings, our concept of artificially creating multi-DOF signal patterns derived from single-DOF ones, so that during training, the user is only required to produce single-DOF signals. If the procedure we propose succeeds, the system will also be able to automatically recognise multi-DOF patterns. This procedure is not a new ML method; rather, it enhances the training data set with novel, synthetic signals and target values. The enhanced data set can then be used by any ML method of choice. The procedure works by linearly combining single-DOF activation signals in the input space, under the strong assumption that multi-DOF activations are represented by weighted, linear combinations of single-DOF ones. We call this procedure *Linearly Enhanced Training* or LET.

Two psychophysical experiments are described in this paper. In the first one we assess the offline feasibility of the approach, checking how well multi-DOF activation patterns can actually be approximated by LET-combined single-DOF ones. This experiment serves the purpose of determining model parameters for the LET procedure and therefore creating a model that is uniform across all subjects and potential future users. In the second experiment, we engage more subjects in an online multi-DOF enforcement task, after the system has been calibrated on single-DOF activations only. Both experiments show promising results. This work extends and to some extent completes the preliminary results published by Castellini et al. [8].

### Related work

Given the nature of s/p prosthetic control, the simultaneous activation of several DOFs of the prosthesis must correspond to simultaneous *muscle* activation. For instance, simultaneously flexing the index and little finger involves, to some extent, the simultaneous activation of the muscles of the forearm (or, at a smaller level, of the motor units), which flex the index finger and the little finger separately. As a result, it is believed that the signal corresponding to the multi-DOF activation is a weighted combination of the signals corresponding to the single-DOF activations involved in the multi-DOF one. In 2009, Jiang et al. [9] proposed a completely linear model of the sEMG signal starting from the neural muscle synergies as represented by the spiking trains in the motor neurons (where the term *simultaneous and proportional control* first appeared). A linear decomposition of the sEMG signal is used to extract single-DOF activations for a prosthetic wrist. However, the single finger cannot be controlled linearly [10] so a more complex model is required.

It is already known to some extent that multi-DOF activation signals somehow lie “in between” of single-DOF activations in the sEMG space. Amsüss et. al. [11] for example present a decision method, in which the distance between the current signal and a set of previous, single-DOF signals is used to determine whether the subject is enforcing a single- or multi-DOF activation. The larger the distance, the more likely it is that the activation is multiple. The idea of a linear decomposition of sEMG signals has been applied in several studies. Yatsenko et al. [12] apply a PCA-based technique to separate combined wrist and hand activations. This technique yields promising results and to a large extent is able to separate these activations. Furthermore, Nagata et al. [13] have conducted a preliminary experiment with only two subjects to separate the activations by the Canonical Discriminant Analysis. The results can be seen as an early proof of concept.

## Materials and Methods

Oral and written descriptions of the experiment were provided to the subjects. After all questions were answered a written consent form was signed by all participants. These experiments are compliant with the World Medical Association’s Declaration of Helsinki, regarding the ethical principles for medical research involving human subjects, last version, as approved at the 59th WMA General Assembly, Seoul, October 2008. Data collection from subjects was approved by the institutional board for protection of data privacy and by the work council of the German Aerospace Center. A physician is part of the council that approved the experiments.

In the following we describe two experiments to establish the LET procedure as a s/p control method. Experiment 1 is concerned with an offline comparison of different training methods, including the full data set containing single- and multi-DOF activations (from now on referred to as the multi-finger or MF data set), the LET-augmented data set and a data set containing only single-DOF activations (from now on referred to as the single-finger or SF data set). A further goal is the determination of a set of parameters for the LET procedure and our ML algorithm that generalises well to unknown users. Experiment 2 is a goal-oriented online task, that serves as a validation of the LET-augmented data set as a s/p control method.

We shall continue with a description of the LET procedure in more detail, followed by a description of the ML method.

### The LET procedure

In the following, $X_{i}\in R^{n\times d}$ denotes the matrix of sEMG data gathered for one particular activation *i*. The dimensionality of the sEMG signal, e.g. the number of sEMG electrodes, is represented by *d* while *n* represents the number of samples. *d* in our case is fixed to 10, *n* on the other hand varies depending on the amount of data gathered. Each *d*-dimensional row in ***X***_*i*_ can be seen as a data point in the *d*-dimensional sEMG space. We therefore refer to ***X***_*i*_ as a data cluster. The data cluster $X_{ij}\in R^{n\times d}$ is an extension of this notation and represents a multi-DOF activation. For example, with *i* = In and *j* = Li, ***X***_In_ contains all the sEMG data gathered during an index finger (In) flexion and ***X***_Li_ all the sEMG data gathered during a little finger (Li) flexion. Furthermore, ***X***_In,Li_ contains all the sEMG data gathered during a combined index and little finger flexion.

This leads us to the central assumption of the LET procedure

$$X_{ij}\approx F\left(X_{i},X_{j}\right)$$ (1)

where F is a function modelling the simultaneous activation of single-DOFs *i* and *j*. The assumption is that by using F the multi-DOF activation can be approximated with sufficient accuracy using only the single-DOF activations that compose it. For example, let’s assume the multi-DOF activation that we want to approximate is the simultaneous flexion of the index and little finger. Thus, by gathering the sEMG data of the single-DOF activations, index and little finger flexion, and by using a model function F we can approximate the sEMG data of the combined flexion of the index and little finger. In case this approximation is sufficiently precise, a ML algorithm trained on the artificial data will be able to predict the simultaneous flexion of the index and little finger correctly. Most importantly, the algorithm is able to do so without the need of performing the multi-DOF activation in the training phase.

Furthermore, to train a ML algorithm we need target values or ground truth for each sEMG data point. We use the so-called “realistic” approach outlined by Gijsberts et al. [10], where the target values are not sensor data such as fingertip forces, but are rather the activation of a stimulus ranging from 0 to 1. In our case we present a visual stimulus of a virtual hand to our subject, where full flexion of a finger denotes a target value of 1 and no flexion a target value of 0. An additional feature of the realistic approach is the fact that training occurs only on full flexion/activation and on no flexion/activation; denoted *on-off* training. Intermediate values are not trained explicitly, but can be interpreted by an appropriate ML algorithm and hence allow the prediction of any intermediate value, which is required in terms of s/p control.

Therefore, we can formulate the training data set for a particular single-DOF activation *i*, ***D***_*i*_, as follows:

$$D_{i}=\left\{\left(X_{0},0\right),\left(X_{i},1\right)\right\}$$ (2)

where ***X***_0_ denotes the data cluster obtained when resting (no activation) with the target value 0; ***X***_*i*_ is the data cluster of the activation of single-DOF *i* with target value 1 for full activation.

We can extend this notation by a further DOF *j* resulting in the following formulation for the training data.

$$D_{ij}=\left\{\left(X_{0},\left(0,0\right)\right),\left(X_{i},\left(1,0\right)\right),\left(X_{j},\left(0,1\right)\right)\right\}$$ (3)

We can see that only one DOF is active at a time, while the other remains silent. Training a ML algorithm on training data ***D***_*ij*_ does not allow the user to perform a simultaneous activation of both DOFs *i* and *j*, as this is not part of the training data.

As mentioned earlier, we aim to approximate multi-DOF activations by using only single-DOF activation data, and in turn use this approximation to extend the training of a ML algorithm. Therefore, the LET procedure represents the augmentation of training data for a ML algorithm in terms of the addition of the artificially created data clusters.

$$D_{ij}^{\text{LET}}=\left\{\begin{array}{c} \left(X_{0},\left(0,0\right)\right),\left(X_{i},\left(1,0\right)\right),\left(X_{j},\left(0,1\right)\right),\\ (F\left(X_{i},X_{j}\right),\left(1,1\right)), \end{array}\right\}$$ (4)

In the equation presented above the LET procedure enhances the training data set ***D***_*ij*_ by the term $\left(F\left(X_{i},X_{j}\right),\left(1,1\right)\right)$. This represents the artificial data cluster created by using a model function F and the single-DOF data clusters ***X***_*i*_ and ***X***_*j*_. Therefore $D_{ij}^{\text{LET}}$ stands for a training data set, which allows the user to perform the single-DOF activations *i* and *j*, as well as the multi-DOF activation that is a combination of the single-DOF activations *i* and *j*.

We have found that in the *d*-dimensional space of the sEMG signal the multi-DOF data can be well approximated by linear combination of the single-DOF sEMG data. Therefore, we formulated two different model-functions, namely the single-*α*-model function $F_{1}$ and the multi-*α*-model function $F_{m}$. The model functions are named after the number of parameters that can be tuned in the approximation of the multi-DOF cluster.

$$F_{1}\left(X_{i},X_{j}\right)=\alpha_{ij}\cdot \left(X_{i}+X_{j}\right)$$ (5)

$$F_{m}\left(X_{i},X_{j}\right)=\alpha_{ij}^{i}\cdot X_{i}+\alpha_{ij}^{j}\cdot X_{j}$$ (6)

In case of $F_{1}$ there is only a single parameter, while in case of $F_{m}$ there is one parameter per single-DOF involved in the multi-DOF activation. The *α*-parameters can be determined given data from single- and multi-DOF activations. Since both model functions can be seen as linear projections onto a lower dimensional space, the *α*-parameters can be interpreted as the weights, which can be determined by minimising a distance measure, in our case the Euclidean distance. To clarify, in the case of the single-*α*-model we confine the search for the best approximation to anywhere along the vector sum of the sEMG data of the single-DOF activations involved in the multi-DOF activation. On the the other hand, for the case of the multi-*α*-model, the confinement is relaxed. We search on the hyperplane spanned by the sEMG data of the single-DOF activations involved in the multi-DOF activation.

To find the optimal *α*-parameter we minimise the Euclidean distance to the real data cluster of the multi-DOF activation with respect to *α*. We use the centre points of the respective data clusters ${\bar{X}}_{i}\in R^{1\times d}$, which are obtained by averaging over the *n* samples of a data cluster. This results in the following expressions for both single- (Eq 7) and multi-*α*-model functions (Eq 8)

$$\alpha_{ij}=\frac{{\bar{X}}_{ij}\cdot {\left({\bar{X}}_{i}+{\bar{X}}_{j}\right)}^{T}}{\left({\bar{X}}_{i}+{\bar{X}}_{j}\right)\cdot {\left({\bar{X}}_{i}+{\bar{X}}_{j}\right)}^{T}}$$ (7)

$$\alpha_{ij}={\left({X_{ij}}^{T}\cdot X_{ij}\right)}^{-1}\cdot {X_{ij}}^{T}\cdot {{\bar{X}}_{ij}}^{T}$$ (8)

with the *α*-parameters of the multi-*α*-model arranged in a vector $\alpha_{ij}={\left[\alpha_{ij}^{i}\;\alpha_{ij}^{j}\right]}^{T}$ and the “basis” vectors of the multi-*α*-model arranged in a matrix $X_{ij}=[{{\bar{X}}_{i}}^{T}\;{{\bar{X}}_{j}}^{T}]$. For the inversion to be valid, it must be ensured that the dimensionality of the sEMG signal is equal to or higher than the number of single-DOF activations involved in the multi-DOF activation.

### Machine learning algorithms

In order to evaluate the effectiveness of the LET procedure, it is necessary to check that the LET-augmented training sets actually produce better prediction models when employed to train a machine learning method. Comparison of different ML approaches is not the main focus of this paper, therefore we selected one particular ML method already established as ideal for myocontrol in existing literature, and then compared its accuracy with that obtained by two other standard ML methods.

As our primary method we chose *Ridge Regression with Random Fourier Features* (RR-RFF), which was already successfully employed in myocontrol by Gijsberts et. al. [10]. RR-RFF is essentially a Least-Squares Support Vector Machine [14, 15] in which, instead of the classical Gaussian kernel, a finite-dimensional approximation of it based upon Fourier coefficients is used [16, 17]. This kernel being finite-dimensional implies that the size of the models generated by RR-RFF is independent of the number of samples in the training set. This is of paramount importance in our case, since LET, while keeping the training short for the subject, still generates an augmented training set which grows exponentially with the number of single-DOF activations. Independence of the size of the training set makes RR-RFF optimal for this kind of problems—the size of the models is always dominated by the dimension of the input space. Another advantage of using RR-RFF is that there is essentially only one hyperparameter to be tuned—the standard deviation of the Gaussian kernel *σ* to be approximated. This parameter can be found by performing a one-dimensional grid search.

From previous experience with RR-RFF the range of the *σ*-grid-search was set to *σ* ∈ [0.05, 6.0]. Variable step size was used to account for higher sensitivity at lower values of *σ*, explicitly the step sizes were chosen to be Δ*σ* = 0.05 for *σ* ∈ [0.05, 1.0], Δ*σ* = 0.1 for *σ* ∈ [1.0, 3.0] and Δ*σ* = 0.2 for *σ* ∈ [3.0, 6.0]. We used the normalised root-mean-squared error (nRMSE) as a performance measure in the grid search

$$\text{nRMSE}=\frac{1}{y_{\text{max}}-y_{\text{min}}}\cdot \sqrt{\frac{\sum^{n}{\left(\hat{y}-y\right)}^{2}}{n}}$$

with $\hat{y}$ as the prediction of the ML algorithm, *y* as the target value, *y*_max_ = 1.0 and *y*_min_ = 0.0. In the course of the grid search we determined the *σ*-value with the lowest nRMSE, as well as all *σ*-values with error values up to nRMSE_min_ + 0.05. This was done to avoid incorrect tendencies due to commonly occurring plateau areas around the minima.

The accuracy of RR-RFF was compared offline with that obtained on the same datasets by Ridge Regression alone (RR, see e.g. [18]), a purely linear approach, and a Support Vector Machine (SVM, [14, 15]) with Gaussian kernel.

### Experimental setup

**Fig 1:**
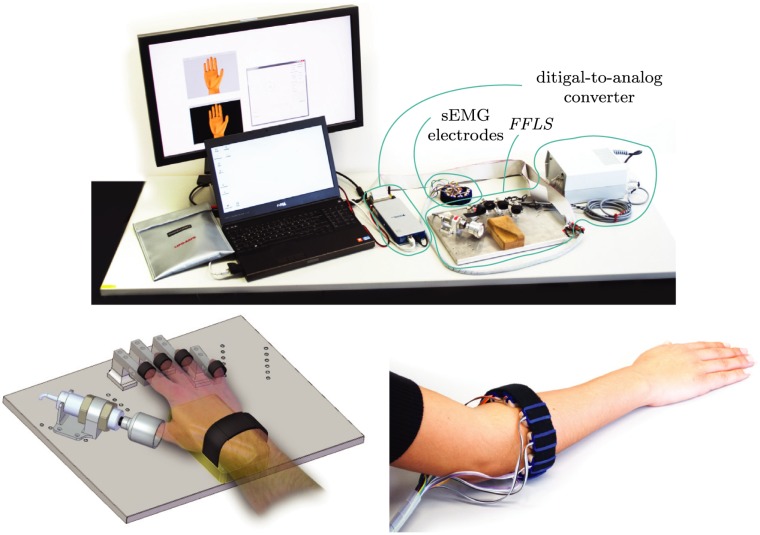
Top: image of the entire setup used in both experiments, including the FFLS and the sEMG electrodes; bottom left: enlarged view of the FFLS with a hand resting in the device (partly reproduced from [19]); bottom right: enlarged view of the sEMG electrodes placed around a subject’s forearm.

Both experiments share a common setup. The main components are depicted in the top panel of Fig 1. Two key features are the *Finger-Force Linear Sensor* (FFLS) and the sEMG electrodes. The FFLS serves as a measurement device for the forces at the fingertips of the right hand of the subject. Furthermore, it immobilises the subject’s hand. This makes each motion performed in experiments 1 and 2 isometric, measuring change in force, but not in length, of a muscle. A subject’s hand resting in the FFLS is depicted on the bottom left of Fig 1. For further information about the FFLS we refer to Kõiva et al. [19]. For acquiring the sEMG signal ten *MyoBock 13E200* sEMG electrodes from *Ottobock HealthCare GmbH* are uniformly arranged around the proximal forearm of each subject using custom built housings for the electrodes on a hook-and-loop strap, shown on the bottom right of Fig 1. This is non-invasive and only measures the electrical potential resulting from muscle contractions that can be perceived on the surface of the skin.

Sensor data is acquired at approximately 62*Hz* using a National Instruments digital-to-analog conversion module (chassis: *NI cDAQ-9181*; card: *NI9205 (DSUB)*) connected by Ethernet to a Windows laptop. The rectified, low-pass filtered and amplified signal of the electrodes is further processed using a 1^st^ order Butterworth filter with a cut-off frequency of 1.5*Hz*. No further feature extraction is performed on the sEMG data. The filtered sEMG signal is then used as an input to the ML algorithm.

Visual information for the subjects and the experimenter was presented on a screen in front of the subject as can be seen in the top panel of Fig 1. All participants were seated in front of the screen, asked to position their right hand on the *FFLS* and adjusted the chair to ensure a comfortable position. The subjects were allowed to rest at any given moment to minimise the influence of fatigue, and allowed to abort the experiment at any sign of discomfort.

### Experiment 1

Ten able-bodied subjects (25 to 42 years of age, two female, eight male) participated in the offline comparison experiment. All participants were right-handed, although this was not a requirement.

**Fig 2:**
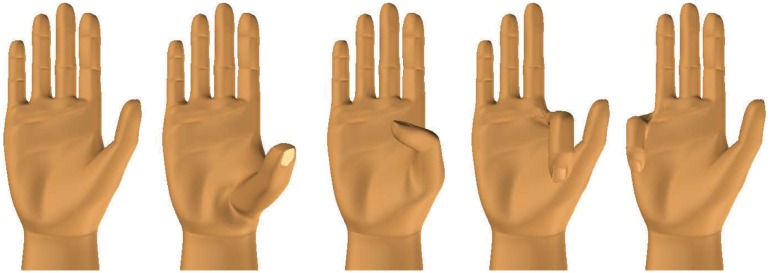
Single-finger activations in experiment 1 from left to right: rest, thumb opposition, thumb flexion, index flexion and little finger flexion.

We chose four different single-finger activations as the controllable DOFs in this experiment, namely thumb opposition, thumb flexion, index flexion and little finger flexion. Thumb opposition refers to a rotational motion of the thumb towards the little finger. All four activations are depicted in Fig 2. All subjects were asked to perform a series of 13 different single- (SF), double- (DF), triple- (TF) and quadruple-finger (QF) activations in increasing complexity. The routine can be found in Table 1. The subjects were asked to maintain each activation for 4.0*s*. Each activation was followed by a resting phase with no fixed duration. The next activation was performed when the subject felt ready. In most of the cases this occurred immediately. Each subject was asked to perform 5 repetitions of these 13 activations, which took on average 19′4*s* ± 2′.

**Table 1:** Routine of experiment 1 in chronological order depicting the DOFs active in each activation.

#	Th. Op.	Th. Fl.	In. Fl.	Li. Fl.	abbreviation
1	1.0	-	-	-	SF1
2	-	1.0	-	-	SF2
3	-	-	1.0	-	SF3
4	-	-	-	1.0	SF4
5	1.0	1.0	-	-	DF1
6	1.0	-	1.0	-	DF2
7	1.0	-	-	1.0	DF3
8	-	1.0	1.0	-	DF4
9	-	1.0	-	1.0	DF5
10	-	-	1.0	1.0	DF6
11	1.0	1.0	1.0	-	TF1
12	-	1.0	1.0	1.0	TF2
13	1.0	1.0	1.0	1.0	QF1

In order to ensure a comparable level of muscle activation across all activations and subjects we used the *FFLS* to modulate the force of each activation. In the first repetition the subjects were asked to perform the 13 activations with the maximum force possible. This was then followed by four repetitions of all 13 activations at 40% of the maximum force of each individual activation. By doing so we ensure that single- and multi-DOF activations are performed at the same fraction of the force relative to the maximum force level of that activation. 40% of the maximum voluntary contraction force was used as the input force for our experiments in order to avoid the degenerative effect of fatigue on the sEMG signal quality. At this level of force one can comfortably perform a number of tasks without any effects of fatigue. Therefore, “1.0” activation in Tables 1 and 2 corresponds to a force level of 40% of the maximum force. It does not correspond to 100% of the maximum force, although an activation of “1.0” will be referred to as *full activation* in the following.

**Table 2:** Sequence of the 18 different activations of experiment 2.

#	Th. Fl.	In. Fl.	Li. Fl.
1	0.5	-	-
2	1.0	-	-
3	-	0.5	-
4	-	1.0	-
5	-	-	0.5
6	-	-	1.0
7	0.5	0.5	-
8	0.5	1.0	-
9	1.0	0.5	-
10	1.0	1.0	-
11	0.5	-	0.5
12	0.5	-	1.0
13	1.0	-	0.5
14	1.0	-	1.0
15	-	0.5	0.5
16	-	0.5	1.0
17	-	1.0	0.5
18	-	1.0	1.0

Visual feedback of the current and desired level of force was provided to the subject. The latter four repetitions were used for the offline evaluation, including a comparison of different training methods and a parameter optimisation.

The performance of LET-augmented training data sets is compared to the performance of different types of training sets, either single-finger data (SF) only, or training sets on both single- and multi-finger data (MF). Following the notation of, Eq 4, this results in the following expressions

$$D_{ij}^{\text{SF}}=\left\{\left(X_{0},\left(0,0\right)\right),\left(X_{i},\left(1,0\right)\right),\left(X_{j},\left(0,1\right)\right)\right\}$$ (9)

$$D_{ij}^{\text{MF}}=\left\{\begin{array}{c} \left(X_{0},\left(0,0\right)\right),\left(X_{i},\left(1,0\right)\right),\left(X_{j},\left(0,1\right)\right),\\ (X_{ij},\left(1,1\right)), \end{array}\right\}$$ (10)

For this comparison a 4-fold repetition-wise cross-validation was used. The number of folds is equivalent to the number of repetitions in the underlying data. Due to filtering and the nature of EMG signals a sample in a time series is closely correlated to the following and preceding sample. Therefore, we only consider samples of different repetitions to be independent from one another and only use entire repetitions as one fold.

Please note that the concepts of the SF, LET and MF training methods are so far independent of the underlying data. These three methods of combining training data can be performed for any of the nine multi-finger activations from Table 1. Further please note that the performance of any of these training methods will be tested against data containing single- and multi-finger activation data. The testing data therefore corresponds to the ***D***^MF^ data set.

With this in mind, we can expect that the MF data set performs the best. Both the training and the testing data of the ML algorithm contains the actual single- and multi-finger sEMG data collected. This is the lower bound for the error. The upper bound is represented by the performance of the SF training data set. The SF data set is also tested against the actual single- and multi-finger sEMG data. Therefore, if the LET-augmented data set performs worse than the SF data set, the LET procedure is fundamentally flawed. We use the nRMSE as an error measure.

In order to investigate the difference between the training methods, ten different training sets were created. Nine of the ten training sets contain only the training data from one of the nine multi-finger activations gathered in experiment 1 (as well as the corresponding single-finger activations). Hence, the training data sets were created as shown in Eqs 4, 9 and 10. Furthermore, a tenth data set was created that contains the three double-finger activations used in experiment 2 (as well as the corresponding single-finger activations).

The sEMG data gathered from the subjects was furthermore used to optimise the hyperparameter *σ* of the ML algorithm and determine the *α*-parameters for both the single- and multi-*α*-model functions by applying Eqs 7 and 8. This was done in two different ways. For the first modality, i.e. the *individual* model, the *α*-values were determined individually for each subject based on the data of said subject. While for the second modality, i.e. the *general* model, the *α*-values were determined using the data from all but the current subject. The *general* model resembles experiment 2 more closely, where the *α*-parameters were taken from experiment 1 and not determined for each subject individually. Hence, we have four different LET modalities, i.e. LET1_ind_, LET*m*_ind_, LET1_gen_ and LET*m*_gen_, which were compared to the modalities SF and MF.

### Experiment 2

For the validation experiment we had 11 participants (21 to 42 years, two female, nine male). Of these 11 subjects three had already participated in the previous experiment. Their respective data was excluded from the optimisation process in their individual experiment. The model parameters were therefore based on thedata of the remaining nine subjects from experiment 1.

For the online validation we chose a reduced set of DOFs, namely thumb flexion, index flexion and little finger flexion. Based on these three DOFs the subjects were asked to perform all of the single- and double-finger activations at two different levels of intensity, which resulted in 18 different activations. We chose to only test two levels of activation, since the number of combinations for more levels of activation will grow exponentially and therefore increases the duration of the experiment beyond a bearable level for the subjects. These activations are summarised in Table 2. We validate our model at two different levels of activation, since we claim that not only simultaneous activation of DOFs is possible using the LET-augmented data set, but proportional activation as well. “Proportional” stands for any intermediate activation between zero and full activation.

In this experiment we used the LET-augmented data set with the optimal parameters from experiment 1. Therefore, we only need to gather single-finger sEMG data from the subjects in the training phase. The same force modulation using the FFLS as in experiment 1 was used in this experiment as well. In a first repetition the subjects were asked to perform the three single-finger activations with maximum force. The sEMG data from this repetition was neglected and only the following three repetitions at 40% of the respective maximum forces were used to create the LET data and to train the ML algorithm. For clarification, the subjects were only asked to perform activations 2, 4 and 6 from Table 2. ML training was only done for activation numbers 2, 4, 6, 10, 14 and 18. The remaining 12 combinations are covered by the s/p control algorithm and explicitly trained.

After gathering the single-finger sEMG data we first trained the algorithm only with the single-finger data. This was followed by a quick qualitative evaluation to ensure the training data was not corrupted. In case of a successful test, the LET training data was incrementally added. The resulting data set corresponds to the LET-augmented training data set. In this pre-test six participants performed satisfactorily on the first attempt, two required two attempts and two required four attempts. One participant did not achieve a satisfactory result on the first day, but returned on the second day to successfully perform the qualitative evaluation on the first try. The training and pre-testing procedure took the subjects on average 6′50*s* ± 4′48*s*.

A goal-oriented validation experiment was performed consisting of three repetitions of the 18 different activations from Table 2 in random order. The subjects received visual feedback of the ML algorithm prediction via a digital hand model. The task was to match the current prediction to another digital hand model acting as a stimulus. With a tolerance of ±0.25 on the target value, the size of the targets was approx. 3.125% of the entire working space. Depending on the dwelling time *t* on the target the attempt was either counted as a success (*t* ≥ *t*_*s*_), an overshoot (0*s* < *t* < *t*_*s*_) or unreachable (*t* = 0*s*) with the success time *t*_*s*_ = 1.5*s* during the attempt duration *t*_*d*_ = 30*s*. The online test of three repetitions of the 18 activations from Table 2 took the subjects on average 18′6*s* ± 5′3*s*.

For evaluating the performance in the online experiment we chose four different measures. Two of these measures are characteristic rates such as the success rate (SR) and percentage of unreachable attempts (UA)

$$SR=\frac{n_{s}}{n_{\text{total}}}\times 100\%\;UA=\frac{n_{u}}{n_{\text{total}}}\times 100\%$$ (11)

with *n*_s_ the number of successful attempts, *n*_u_ the number of unreachable attempts and *n*_total_ the total number of attempts. Furthermore, we evaluated the time it took a subject to complete a task successfully, starting from the appearance of the stimulus and ending after staying on the target for the duration *t*_s_. This will be called *task completion time* (TCT). The overshoots were evaluated in terms of the *longest stable time* (LST). This represents the longest duration a subject was able to remain in the target area. This measure is bounded by the success time *t*_s_.

These measures are similar to the ones that have been used to evaluate the online experiment by Jiang et al. [20].

### Statistical analysis

For the statistical analysis we used the *Statistics Toolbox* provided in the computing environment *MATLAB* from *The MathWorks, Inc.* as well as the statistical tools provided in the programming language *R*. For performing a comparison between two samples Student’s *t*-test has been used [21]; the one-way ANOVA [22, 23] was chosen for comparing more than two samples.

To evaluate experiment 2 we performed a multivariate two-way ANOVA [24, 25]. The two factors were the number of DOFs used (single- or multi-finger activation) and the repetitions (1 to 3). The influence of these factors was investigated in terms of the four measures introduced at the end of the previous subsection. This was followed by a reduced comparison in case significance was found.

For all tests the level of significance was set to 0.05. As a post-hoc test we performed the *Tukey*-test to determine the pairwise interactions.

## Results

This section provides the results of the two experiments described in the previous section, in particular the offline comparison of different training data sets, the parameter optimisation and the online validation.

### Results of experiment 1

**Fig 3:**
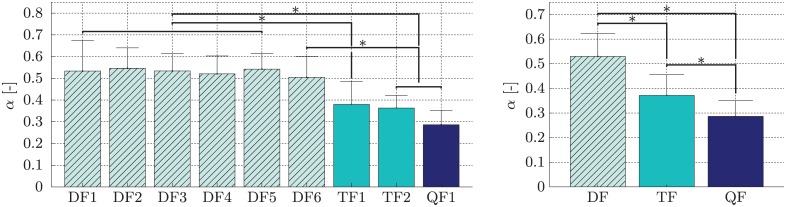
Left: means and standard deviations of *α*-values across subjects for all combinations for the single-*α*-model; right: means and standard deviations of *α*-values across combinations with the same number of fingers and across subjects for the single-*α*-model. (Brackets without asterisks indicate grouping. All members of a group share the same interactions).

**Fig 4:**
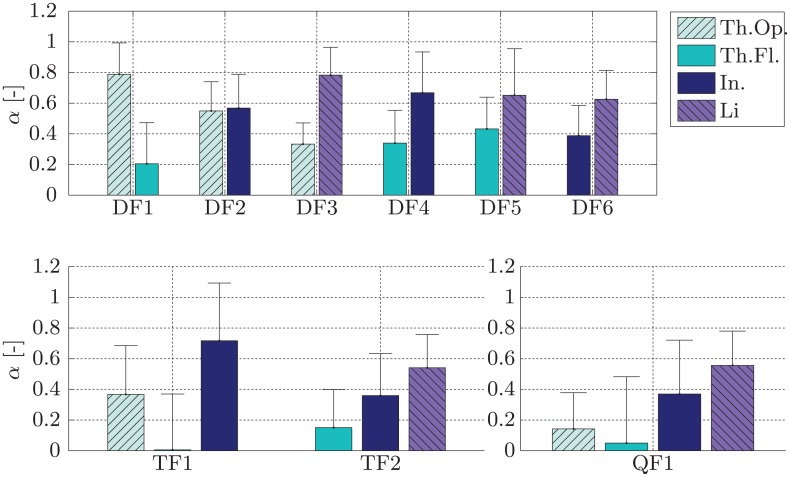
Means and standard deviations of *α*-values across subjects for all combinations for the multi-*α*-model.

**LET-parameter optimisation** Using the single- and multi-finger sEMG data gathered in experiment 1 we were able to determine the *α*-parameters for both model functions. The mean and standard deviation across subjects for the *α*-parameters of the single-*α*-model for the nine multi-finger activations in Table 1 are shown in Fig 3. For the multi-*α*-model the respective means and standard deviations across subjects for all 22 parameters can be found in Fig 4. The resulting parameters of the single-*α*-model allow for a further simplification of the model. The *α*-values can be grouped by the number of fingers involved in the multi-finger activation (*α*_DF_ = 0.5301 ± 0.0941, *α*_TF_ = 0.3714 ± 0.0841 and *α*_QF_ = 0.2863 ± 0.0645).

In Fig 3 on the left we can see that the *α*-values of the double-finger activations are significantly different from those of the triple- and quadruple activations, with the exception of the interaction between *α*_DF6_ and *α*_TF1_, which is not significant (*p* = 0.074). Representing the *α*-values grouped by the number of fingers shows that all values are significantly different from each other with *p* = 0.042 for the interaction of *α*_TF_ and *α*_QF_ and *p* < 10^−3^ for the remaining two interactions. This can be seen in Fig 3 on the right. A table containing the *p*-values for all interactions of the *α*-values of the single-*α*-model can be found in S1 Table in the Supporting Information. These values are based on the post-hoc *Tukey*-test, which was performed after a one-way ANOVA showed significant difference for the *α*-values of the single-*α*-model. In case of the multi-*α*-model no statistical evaluation was performed.

**Method comparison** A comparison between different training data sets has been performed for evaluation of the overall performance of the LET procedure.

**Fig 5:**
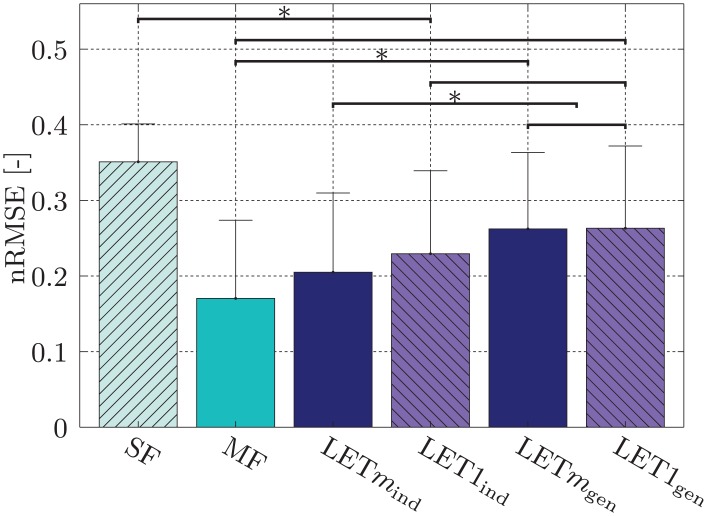
RR-RFF: means and standard deviations of the nRMSE across all subjects and across the ten training sets for different methods. (Brackets without asterisks indicate grouping. All members of a group share the same interactions).

The averaged results for the six methods introduced in Section *Materials and Methods* can be found in Fig 5 with the mean and standard deviation across all subjects and the ten training sets. A one-way ANOVA showed significant interaction between the aforementioned six methods. Following the ANOVA we performed the *Tukey*-test to determine the pairwise interactions. Table 3 shows the *p*-values of the pairwise interactions of the different training methods. We can see that there is a difference in performance between the two *individual* models, although it is not statistically significant (*p* = 0.50), while in the *general* case there is virtually no difference in the error measure. As stated before the *general* model more closely resembles experiment 2. Since no difference can be found between LET1_gen_ and LET*m*_gen_ (*p* ≈ 1.00), we decided to test only one modality in experiment 2 in order to reduce the time of the experiment for the user. We chose the less complex single-*α*-model, since the additional parameters of the multi-*α*-model don’t seem to lead to a better performance in the online case.

**Table 3:** Pairwise *p*-values for the interaction of the nRMSE-values of the different training methods based on the post-hoc *Tukey*-test.

	SF	MF	LET1_ind_	LET*m*_ind_	LET1_gen_	LET*m*_gen_
SF		< 10^−3^	< 10^−3^	< 10^−3^	< 10^−3^	< 10^−3^
MF	< 10^−3^		< 10^−3^	0.13	< 10^−3^	< 10^−3^
LET1_ind_	< 10^−3^	< 10^−3^		0.50	0.15	0.17
LET*m*_ind_	< 10^−3^	0.13	0.50		< 10^−3^	< 10^−3^
LET1_gen_	< 10^−3^	< 10^−3^	0.15	< 10^−3^		1.00
LET*m*_gen_	< 10^−3^	< 10^−3^	0.17	< 10^−3^	1.00	

**Machine learning algorithm comparison** In order to show the capability of the LET procedure we used this method in combination with two common ML algorithms in the community, namely Ridge Regression (RR) and Support Vector Machine (SVM). The same calculations that have been performed for RR-RFF have been performed for RR and SVM. The resulting nRMSE values can be found in Table 4.

**Table 4:** Means and standard deviations of the nRMSE for three ML algorithms and six different training methods.

	RR	RR-RFF	SVM
SF	0.3456 ± 0.0743	0.3510 ± 0.0501	0.3345 ± 0.0491
MF	0.2598 ± 0.0902	0.1704 ± 0.1033	0.1547 ± 0.0882
LET*m*_ind_	0.2994 ± 0.0670	0.2050 ± 0.1048	0.1847 ± 0.0845
LET1_ind_	0.3191 ± 0.0788	0.2294 ± 0.1098	0.2054 ± 0.0856
LET*m*_gen_	0.3132 ± 0.0740	0.2625 ± 0.1014	0.2311 ± 0.0821
LET1_gen_	0.3204 ± 0.0782	0.2634 ± 0.1090	0.2336 ± 0.0870

### Results of experiment 2

**Fig 6:**
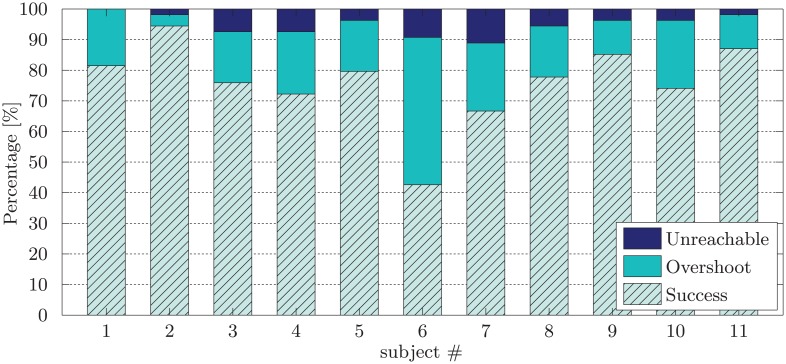
Overview of successful attempt, overshoots and unreachable attempts for all subjects.

The results of the task-oriented experiment separated by subject can be found in Fig 6. For each subject the percentages of successful attempts, overshoots and unreachable attempts have been depicted. On average 76.09 ± 12.84% of the attempts were successful.

**Fig 7:**
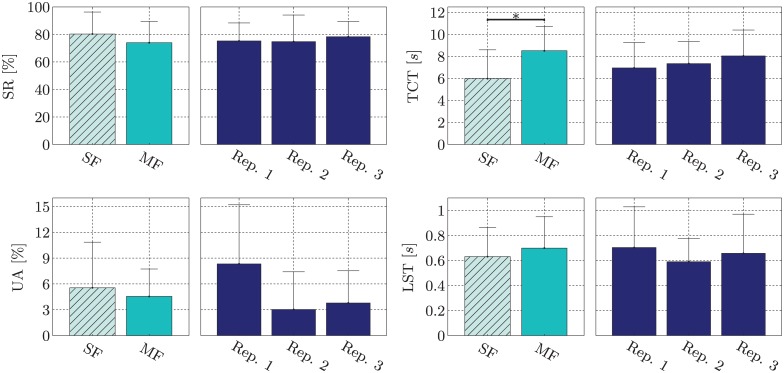
Means and standard deviations across subjects for the SR, TCT, UA and LST grouped by number of finger involved in the activations and by repetition.

Furthermore, we grouped the tasks into single- and multi-finger tasks as well as by repetition. For this grouping the mean and standard deviation across all subjects for the success rate (SR), task completion time (TCT), percentage of unreachable attempts (UA) and longest stable time (LST) can be found in Fig 7.

Multivariate, two-way ANOVA was performed on the four performance measures investigating the two factors *number of DOFs involved in an activation* (single- or multi-finger activation) and *repetition* (first, second or third). The first factor, *number of DOFs involved in an activation*, was significant (*p* = 0.004), while the second factor, *repetition*, was not (*p* = 0.231). For the first factor, further pairwise comparisons were performed showing that only the TCT exhibits significant difference between single- and multi-finger activations (*p* = 0.004). Since we consider SR to be the most informative performance measure, we would like to highlight that the difference between SR_SF_ and SR_MF_ was not significant (*p* = 0.203).

## Discussion

### Experiment 1

The comparison of the two *general* LET-methods shows that both perform significantly worse than the MF data sets; this was to be expected since MF is the ideal case in which the machine knows exactly what the multi-DOF activations look like, whereas LET is an approximation of that situation. However, none of the two *general* LET methods proves to outperform the other. In the *individual* case, performance of the multi-*α*-model results in an overall lower error, although this performance difference is not significant (*p* = 0.50). The *general* case represents a cross validation in order to reduce the influence of overfitting of the model to the user. This is a more realistic case under the assumption that the *α*-values have been determined once on the data of a sample population and eventually are used without the need of individual adaptation to the user. The performance difference present in the *individual* case is not evident in the *general* case. This could be evidence of overfitting, when the *α*-values are determined *individually* for each user using the multi-*α*-model.

A further, very interesting remark is that there is little difference between the *α*-values of activations with the same number of fingers involved for the single-*α*-model; for instance, the *α*s for all two-DOF activations are in the same range. Thus, we can simplify our model from nine parameters to only three, one for double-, one for triple- and one for quadruple-finger activations. We claim that these parameters combined with *σ*^LET1^ (see Supporting Information) are suited to allow for s/p control. These parameters were validated in experiment 2.

The comparison among RR, RR-RFF and SVM shows that RR-RFF and SVM demonstrate a higher accuracy than RR, with SVM being even more accurate than RR-RFF. These results are expected, and in line with those obtained by Gijsberts et. al. [10]. The models generated by RR is linear, which can result in larger errors. The results obtained by RR are used as the baseline. RR-RFF is a finite-dimensional approximation of SVM, both of which are non-linear. Therefore, we expect that the accuracy of RR-RFF somehow lies in between that of RR and SVM.

### Experiment 2

Overall, 76.09% of all tasks in experiment 2 were successful. We have found no evidence of significant difference between the SR of single-finger activations and the SR of multi-finger activations. This is the central point of LET. In the training phase the participants only performed single-finger activations, whereas the multi-finger activations were only introduced in terms of artificial model data. Still, the average performance of SF and MF, with 80.30% and 73.99% respectively, are comparable. The significantly higher TCT for multi-finger activations, compared to single-finger activations, implies that multi-finger activations were more difficult to perform. This may be due to the fact that two fingers instead of one finger have to be controlled precisely. Furthermore, not all performance measures indicate a better performance on single-finger activations. The UA rate shows that more single-finger activations were unreachable than multi-finger activations. Regarding the LST there is a slight increase from single- to multi-finger activations. This could be interpreted as slightly improved stability with multi-finger activations.

Additionally, we see evidence of two further emerging effects. Firstly, we can see an increase of TCT across the repetitions. This could indicate fatigue, possibly due to uncomfortable or unusual finger activations. Secondly, the UA indicates that there is some form of reciprocal learning effect present. While the first repetition showed high UA rates, the second and third repetition show considerably lower UA rates. One can assume that the subject adapted to the new control methods.

### General discussion

The LET procedure can be summed up as follows: after a subject has produced single-DOF patterns, LET augments the training set by linearly combining them into artificial signals representing an approximation of the true multi-DOF activations. After the LET-augmented training set is built, any ML method of choice can be used to associate both single- and multi-DOF activations to the correct target values. However, in practice some constraints must be posed on the ML method itself, which is discussed in more detail below. The aim of this study was to investigate whether the training procedure could be significantly shortened for the subject, since the subject only needs to produce single-DOF activation signals, which can be used for multi-DOF activations as well.

The experimental results indicate that the LET procedure can effectively lead to correct interpretation of multi-DOF activations by a system trained on single-DOF only. In particular, the first experiment aimed at checking whether common parameter values could be found for all subjects. We engaged the subjects in an offline experiment to perform multi-DOF activations in addition to single-DOF ones. Based on that data, we were able to evaluate the *α* parameters. The results indicate that the optimal *α*s are to a large extent invariant across subjects, and more so when the simpler single-*α*-model is employed. Since the difference between this model and the more complex multi-*α*-model is statistically negligible, we recommend the simple model for online application.

The second experiment, in which the subjects were engaged in reaching tasks, confirms that the common parameters found in the first experiment are usable “out of the box”. In this experiment, to train the machine, the subjects *did not perform multi-DOF activations*. Still, an acceptable level of performance was achieved. This is all the more interesting since the performance of ML methods in an online task might be completely independent from the performance obtained by the very same ML methods while evaluated offline [20]. The trend in the community is that of favouring online experiments and “practical” measures of performance such as, e.g., the percentage of successful task completions, rather than abstract measures, e.g. the nRMSE. We believe that LET could be effectively used in a real-life scenario.

In principle, LET can be applied to any kind of multi-DOF prosthesis. One useful case would be the extension of these results to a fully-fledged upper-limb prosthesis gifted with eight to ten DOF, for example a 2-DOF shoulder, a humeral rotator, a prosthetic elbow and wrist and a multi-fingered prosthetic hand. In this case LET would help to automatically generate multi-DOF activations such as, e.g., grasping a mug while flexing the elbow and pronating the wrist, while the user would have trained the prosthesis on single-DOF activations only. In such a case, the patient must be prepared with TMR (Targeted Muscle Reinnervation, see, e.g., [26, 27]); simplifying the training procedure would be highly beneficial in the rehabilitation / training loop, since TMR can require up to several months to achieve the best results.

As already observed, the main limitation of LET consists in the fact that, although the subject training time is reduced, the augmented training set remains exponentially large in the number of single-DOF activations. In this practical setting, this could be detrimental, since many ML methods heavily depend on the number of acquired training samples *n*. The typical example is given by SVM [14], whose time complexity is $O(n^{3})$. To prevent this phenomenon from appearing during the online experiments, we have limited the choice of the ML method to be applied to the LET-augmented datasets to RR-RFF. RR-RFF has the advantage that it does not depend on *n* but only on the dimensionality of the input space. We followed the approach outlined by Gijsberts et al. [10]. A comparison among Ridge Regression, RR-RFF and SVM showed that, as expected, RR-RFF performs like an approximation of an SVM. Nevertheless, let us remark once again that *LET is no novel ML method*, and that other methods could work in this setting just as well.

When we compared the cases of different multi-DOF activations to one another, an interesting phenomenon is observed: *α* seemed to be proportionally bound to the *number of single-DOF activations* involved in each multi-DOF activation considered. This might be the manifestation of a physiological phenomenon described by Li et al. [28]. It has been observed that the maximum force that can be exerted with a single finger in a single-finger task cannot be reached again with that single finger in a multi-finger task. Several hypotheses were formulated by Li et al. [28] to explain this behaviour, among these were synergistic neural inhibition and a neural drive ceiling. We have observed this exact phenomenon in the decrease of the *α*-values, which might be due to the use of the force at the maximal voluntary contraction (MVC) for modulating the sEMG-signals. Since the sEMG-signal is supposed to be linearly correlated to force values for low force levels (up to about 50% MVC [29]), this might be the physiological explanation for the decrease in *α*-values for the increasing number of DOFs involved in multi-DOF activations.

We close the discussion with a final remark on reciprocal adaptation. Some scholars in the myocontrol community decree that online experiments, besides being more useful than offline ones, could produce *better* results than offline evaluations, since there should be some degree of adaptation of the user to the machine, as well as of the machine to the user. As an analogy, controlling a self-powered prosthesis is like learning to ride a motorbike. The more the user wears the device, the more proficient the user becomes at controlling it. In our case, this may have occurred in the second experiment. As opposed to what happened in the first one, in the second experiment the subjects were immediately engaged in a goal-directed task, consisting of tracking a visual stimulus. It is likely that the subjects were adapting their signals to what was required, rather than blindly attempting to produce the multi-DOF activations. We are currently not able to evaluate and/or quantify, whether this phenomenon helps to significantly improve the performance. If confirmed, this effect might be further explored for amputee patients. On the other hand, inducing the user to produce signals that do not match their own original patterns could lead to undesired effects such as, e.g. phantom-limb pain [30]. This issue is subject to future research.

Our immediate future work involves a similar, well-known problem already tackled in literature (e.g. Amsüss et al. [11]) of enabling a trans-radial amputee to control pronation and flexion of a prosthetic wrist (plus opening and closing of the hand). Our study does not deal with that, although the DOFs of the wrist are of great relevance in daily-life activities. We plan to apply LET to this problem, noting that s/p control of a hand and wrist is, in principle, a special case of the class of problems solvable by LET. If proven successful in this case, it could also be possible to fuse s/p control of the wrist with that of the hand, either finger-by-finger or by enforcing a reduced set of predefined grasps.

## Conclusion

In this paper we presented and successfully tested the LET procedure. LET allows the prediction of multi-DOF activations, although the subject only trains the ML algorithm with single-DOF activations. This shortens the training procedure, reducing stress and cognitive burden on the subject him/herself. Our experimental results confirm that LET works both in the offline and online setting and that it can be used by tuning only one parameter. This parameter, called *α*, can be considered invariant across subjects, as shown in the experiments we have carried out. The subject independence makes the parameter potentially suitable to all users.

## Supporting Information

S1 Appendix ML-parameter optimisation. (PDF)

S1 Fig Outcome of the *σ* grid search for a typical subject. (EPS)

S2 Fig Exponential fit of the *σ*_opt.+0.05_ histogram for the LET1 training method. (EPS)

S1 Table Pairwise p-values for the interaction of the *α*-values of the single-*α*-model based on the post-hoc *Tukey*-test. (EPS)

S1 File S1_File.zip: Experimental data. (ZIP)
