# Supplementary material for: The LET Procedure for Prosthetic Myocontrol: Towards Multi-DOF Control Using Single-DOF Activations
Source: PLoS One. 2016 Sep 8;11(9):e0161678. doi: 10.1371/journal.pone.0161678 (PMC5015907; doi:10.1371/journal.pone.0161678)
Supplement: S1 Appendix — (PDF) [file pone.0161678.s001.pdf]

S1 Appendix. ML-parameter optimisation

In order to determine a model that has been optimised to a user, one further parameter, besides the α-parameters, has to be determined. This parameter is specific to the ML algorithm of our choosing and represent the variance σ of the Gaussian kernel used in our ML method. Based on the data gathered in the first experiment a grid search was performed with a 4-fold repetition-wise cross-validation. An example for an average subject can be found in S1 Fig with mean and standard deviation.

The asterisk marks the σ-value will the lowest nRMSE. The σ-values extracted from the grid search were plotted in a histogram and exponential curve fitting was performed on these values for each of the four training methods. Exemplary, the result of the LE1T data can be found in S2 Fig. The maxima of the fitted curves can all be found in the vicinity of 1 ($\sigma^{\mathrm{SF}} = 1.0955$, $\sigma^{\mathrm{MF}} = 0.9795$, $\sigma^{\mathrm{LET1}} = 0.9545$ and $\sigma^{\mathrm{LET}m} = 1.0770$).
